# Thyroid Hormones in Hemolymph of Red King Crabs from the Barents Sea

**DOI:** 10.3390/ani12030379

**Published:** 2022-02-04

**Authors:** Alexander G. Dvoretsky, Elena V. Tipisova, Viktoria A. Alikina, Aleksandra E. Elfimova, Vladimir G. Dvoretsky

**Affiliations:** 1 Murmansk Marine Biological Institute of the Russian Academy of Sciences (MMBI RAS), 183010 Murmansk, Russia; ag-dvoretsky@yandex.ru; 2 N. Laverov Federal Center for Integrated Arctic Research of the Ural Branch of the Russian Academy of Sciences (FECIAR UrB RAS), 163000 Arkhangelsk, Russia; tipisova@rambler.ru (E.V.T.); victoria-popcova@yandex.ru (V.A.A.); a.elfimova86@mail.ru (A.E.E.)

**Keywords:** *Paralithodes camtschaticus*, red king crab, thyroid hormones, triiodothyronine, thyroxine, aquaculture, Barents Sea

## Abstract

**Simple Summary:**

In vertebrates, thyroid hormones, triiodothyronine, and thyroxine play a pivotal role in regulating development, metamorphosis, and metabolism. Recently, the presence of endogenous thyroid hormone signaling has also been reported in invertebrates. We assayed thyroid hormone profiles in the red king crab, an iconic commercially important species, which was introduced into the Barents Sea in the 1960s and now is supporting a large-scale profitable fishery. Both thyroid hormones were detected in the crab hemolymph. Their levels fluctuated significantly depending on the maturity status of the crabs and sampling season. These findings confirm that thyroid hormones are involved in the specific physiological processes of red king crabs and may be used in aquaculture.

**Abstract:**

Only a few studies have reported the presence and levels of thyroid hormones in invertebrates. At the same time, the administration of these hormones has been shown to be a profitable method for inducing growth in crustaceans. Knowledge of thyroid hormone concentrations is required to involve this approach in crustacean aquaculture. For this reason, we examined hemolymph samples of the invasive red king crab *Paralithodes camtschaticus* from the Barents Sea population for the levels of triiodothyronine (T3) and thyroxine (T4). Radioimmunoassays indicated that T3 ranged from 0.26 to 1.55 μg dL^–1^, whereas T4 varied from 52 to 203 μg dL^–1^. Adult crabs demonstrated higher levels of T3 than juveniles, while juvenile crabs had higher levels of T4 in comparison to larger conspecifics. The thyroxine levels were consistent throughout the study period. In contrast, significant seasonal variations were found for T3. In immature crabs, the maximum concentration was found in October, whereas in adult crabs, the highest level of T3 was registered in August. Most likely, these results are explained by changes in physiological conditions of the crabs associated with their molting calendar and migration activity. Our data may have important implications for aquaculture of *Paralithodes camtschaticus* in the Barents Sea.

## 1. Introduction

There are two main thyroid hormones: 3’,5’,3,5-L-tetraiodothyronine (thyroxine), which is nowadays commonly called T4, and 3’,5,3-L-triiodothyronine, T3 [1]. These hormones are composed of two tyrosyl residues linked through an ether linkage and substituted with 4 or 3 iodine residues, respectively. In vertebrates, triiodothyronine is the biologically active hormone, whereas thyroxine, the major thyroid hormone that is secreted from the thyroid gland consisting of follicles, is a precursor or prohormone. Deiodination of T4 leads to production of T3 and reverse T3 (rT3) [2]. Thyroids play a crucial role in regulating early development, metamorphosis, and metabolism in mammals, amphibians, and fish, and most vertebrates are unable to grow and reach their normal adult form without them [3,4,5,6]. Non-vertebrate chordates, such as tunicates, cephalochordates, and the larvae of lampreys have the endostyle, which acts as a thyroid gland homolog [7,8]. Non-chordate invertebrates without thyroid follicles have been shown to accumulate iodo-compounds exogenously from seawater and through diets containing micro- and macroalgae [9]. Heyland et al. [10] suggested the presence of endogenous thyroid hormone signaling in echinoderms and mollusks, which may reflect the existence of iodination processes. There are only a few reports concerning thyroid hormone profiles in invertebrates, including terrestrial gastropods, marine bivalves, and mud crabs [11,12,13]. Because thyroid hormones are able to affect reproduction processes in invertebrates, these substances are considered promising agents in shellfish aquaculture [14,15,16].

The red king crab, *Paralithodes camtschaticus* (Tilesius, 1815), is an example of a well-organized and successful introduction of a non-indigenous species to a new place of habitat to establish a new population for commercial exploration. On the one hand, although some authors reported alterations in species composition, structure, and biomass of benthic communities at some coastal sites of the Barents after the crab introduction [17,18], these changes are not as critical as was predicted and the major fish stocks remained unaffected [19]. On the other hand, *Paralithodes camtschaticus* has become abundant both in the coastal waters and in the open Barents Sea and now its population supports a profitable fishery [19,20,21,22]. In Russia, this fishery was initiated in 2004 and stock indices of *Paralithodes camtschaticus* fluctuated over subsequent years due to range extension of the crabs, overfishing, and climate change [19,20,23,24]. Annual landings have increased from 9187 metric tons in 2018 to 9836 t in 2019 and 10,820 t in 2020 [21,25,26,27]. The total allowable catch for the 2021 fishery season was set at 11,815 t [25]. Moreover, the red crab stock now supports recreational fishing with an annual quota of 100 t [28].

Although many life-history traits of the Barents Sea red king crabs are well-studied [19,20,21,22,23,24,29,30,31,32,33,34,35,36], there is a lack of knowledge on their physiology, especially, hormone profiles in the hemolymph [37,38,39]. Recently, the presence of thyroid hormones has been reported in red king crabs [40], but those data are restricted and more detailed information is required for further research, monitoring, and aquaculture purposes.

For this reason, the aim of our study was to measure concentrations of thyroid hormones in hemolymph of red king crabs from the Barents Sea in relation to their sex, maturity status, and sampling season.

## 2. Materials and Methods

**Figure 1:**
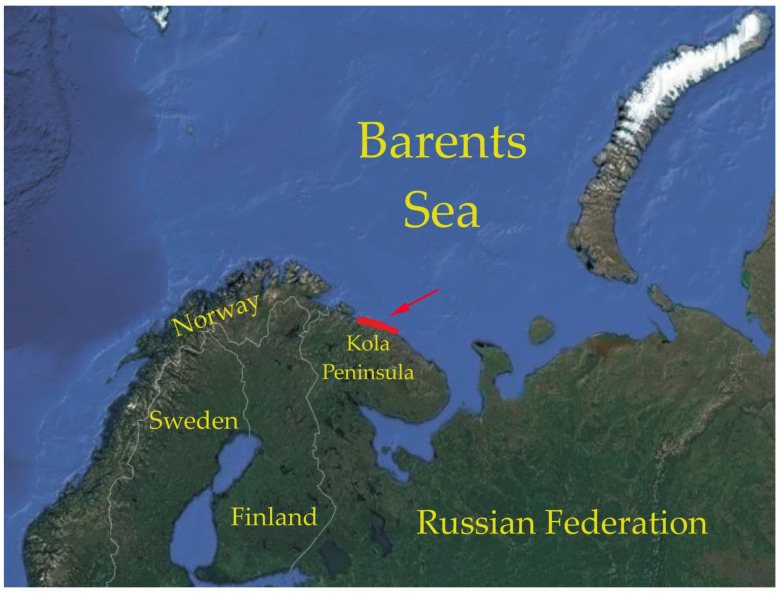
Map of the study area.

Red king crabs were collected by divers at 5–38 m and by using bottom traps at 50–60 m depths in the coastal waters of the Kola Peninsula (Southern Barents Sea, 69.26° N, 33.3° E–69.11° N, 36.1° E, Figure 1) in June and August 2008 and May, July, and October 2009.

After being collected, the crabs were visually inspected for sex and shell condition. Each crab was weighed and its size was measured using calipers across the carapace length (CL, the greatest straight-line distance from the posterior margin of the right eye orbit to the medial-posterior margin of the carapace) [41].

All the crabs used for biochemical analyses had new or old shells (2–20 months post ecdysis) [41]. 

Male red king crabs with CL < 90 mm were considered “juveniles” whereas males with the CL > 90 mm were classified as “adults” according to Dvoretsky and Dvoretsky [36]. This separation corresponds to the results of histological data: the individuals of *P. camtschaticus* larger than 90 mm CL had ripe spermatozoa in their testes in contrast to smaller specimens [39]. The same female groups were separated according to both size-at-maturity data (presence or absence of external eggs on their pleopods) [42] and histological data (mature females had oocyte diameter > 280 μm) [29].

Hemolymph samples (volume 0.6 mL) were taken from the articulating membrane between the dactyl and propodus of the third walking leg using a sterile syringe with a needle, both washed with an anticoagulant containing sodium citrate and EDTA to prevent coagulation of the samples [39]. Each hemolymph sample was placed in a sterile 2-mL plastic tube containing 1.4 mL of 96% ethanol. The samples were frozen and then transported to the laboratory at the Institute of Physiology of Natural Adaptations (Arkhangelsk, Russia) for biochemical analyses. The analyses were performed within 2–4 months after sampling.

Radioimmunoassays were carried out according to the protocols suggested by the manufacturer (A Beckman Coulter Company, Villepinte, France) using Immunotech Total T4 RIA KIT and Immunotech Total T3 RIA KIT. In the case of total thyroxine (TT4), a 20-μL hemolymph sample and 500 μL of ^125^I-labelled T4 were added serially to an antibody-coated tube. In the case of total triiodothyronine (TT3), a 25-μL hemolymph sample and 200 μL of ^125^I-labelled T3 were used in the assays. A tube containing 500 μL of ^125^I-labelled T4 (or 200 μL of ^125^I-labelled T3) was included in every assay to determine the total ^125^I-activity (the sample “T”). Tubes with “total thyroxine” or “total triiodothyronine” were incubated with constant shaking (>280 rpm) at ambient temperature (18–25 °C) for 1 h. At the end of incubation, the radioactivity of samples was determined using a scintillation counter (ARIAN, producer OOO VITAKO, Moscow, Russia). The standard graphs were plotted and the concentrations of total thyroxine or total triiodothyronine were obtained from the standard plot. The results were multiplied by a dilution factor of 3.33. Because thyroxine was observed at concentrations above the established working range for the kit, the hemolymph samples were additionally diluted 2 or 3 times with 95% ethanol and the results were then multiplied by a dilution factor of 2 or 3. The analytical sensitivity of the total thyroxine kit was 1010.1 ng dL^–1^ (13 nmol L^–1^), the working RIA range was 1010.1–31,080 ng dL^–1^ (13–400 nmol L^–1^), and the coefficient of variation between samples was 6.2%. For the total triiodothyronine kit, these levels were 19.5 ng dL^–1^ (0.3 nmol L^–1^), 19.5–780 ng dL^–1^ (0.3–12 nmol L^–1^), and 6.3%, respectively.

Size and weight data were compared between male and female red king crabs and between juveniles and adults using a one-way analysis of variance (ANOVA) for normal data or non-parametric Kruskal–Wallis tests in cases of non-normal data distribution. The same approaches were applied for the levels of thyroid hormones in relation to the sex and maturity status of red king crabs as well as sampling season. When the test was significant, mean values for different groups were compared using the Tukey–Kramer test for mean values or Bonferroni test for medians, and differences were considered significant at *p* < 0.05. Statistical analyses were carried out using Statistica 10. Mean values are presented with standard errors (± SE).

## 3. Results

**Figure 2:**
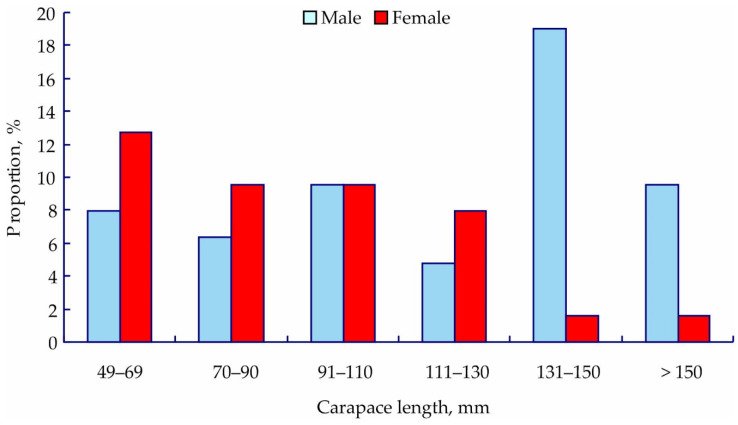
Size–frequency distribution of red king crabs from the coastal Barents Sea.

A total of 23 juvenile crabs (16 females and 7 males) and 40 adult individuals (11 females and 29 males) were captured in the Southern Barents Sea (Figure 2). Their size and weight variations are summarized in Table 1.

Mean carapace length and weight in juvenile males and females were similar (ANOVA, df = 1, F = 0.534, *p* = 0.473 for CL and Kruskal–Wallis test, df = 1, H = 0.599, *p* = 0.447 for weight). The same result was obtained for adult specimens (ANOVA, df = 1, F = 2.027, *p* = 0.155 for CL and Kruskal–Wallis test, df = 1, H = 2.248, *p* = 0.133 for weight) 

**Figure 3:**
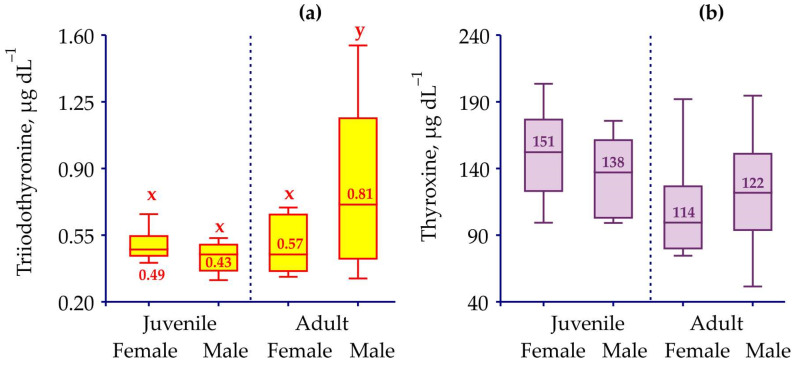
Variations in thyroid hormones in hemolymph of red king crabs (box plots) in relation to their sex and maturity status. Numbers indicate mean levels. Bars with the same letter are not significantly different (*p* > 0.05). (**a**) Triiodothyronine, (**b**) thyroxine.

Biochemical assays have shown the presence of both triiodothyronine and thyroxine in the hemolymph of the red king crabs analyzed. Concentrations of T3 varied from 0.26 to 1.55 μg dL^–1^ (mean, 0.64 ± 0.04 μg dL^–1^), while concentrations of T4 ranged from 52 to 203 μg dL^–1^ (mean, 129 ± 5 μg dL^–1^). These levels demonstrated fluctuations depending on size, sex, and hormones (Figure 3). There were no significant differences between the T3 levels in juvenile males, juvenile females, and adult females, whereas juvenile concentrations of T3 were significantly lower in comparison to adult males (Table 2). 

The adult male and female concentrations did not differ significantly while the combined T3 level in females (0.52 ± 0.04 μg dL^–1^) was significantly lower than in males (0.74 ± 0.06 μg dL^–1^) (Table 2). In general, juveniles exhibited lower levels of T3 than adult crabs (0.47 ± 0.02 vs. 0.74 ± 0.06 μg dL^–1^). The comparisons of T4 levels in male and female red king crabs as well as in adult and juvenile specimens have shown similar concentrations between almost all the combinations of the groups compared, except for juvenile and adult females (Figure 3, Table 2). Contrary to T3, the hemolymph of juvenile crabs contained higher concentrations of T4 than that of adult specimens (147 ± 6 vs. 112 ± 6 μg dL^–1^) (Table 2).

**Figure 4:**
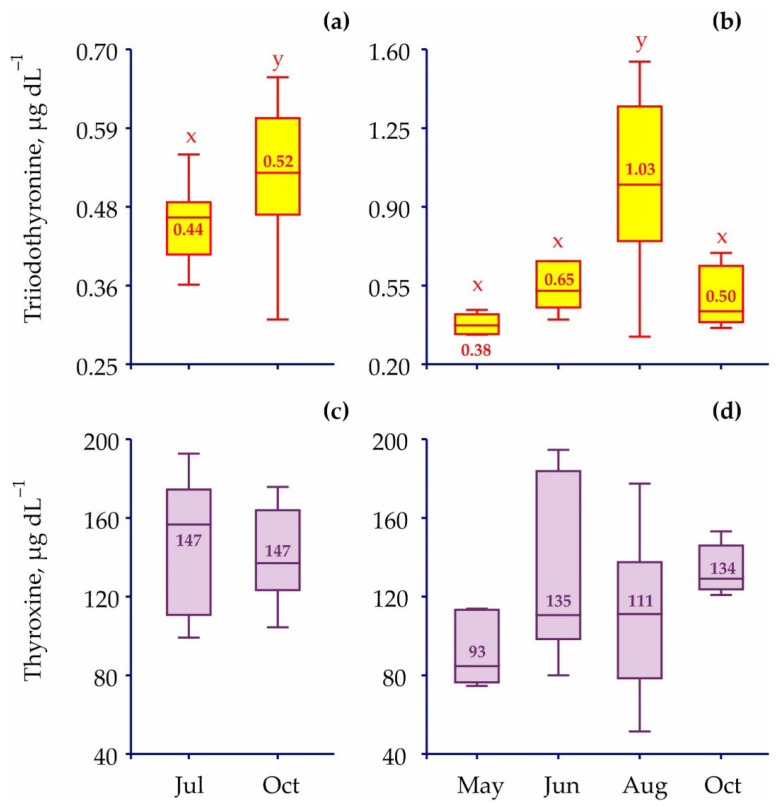
Seasonal variations in thyroid hormones in hemolymph of red king crabs from the coastal Barents Sea (box plots). Numbers indicate mean levels. Bars with the same letter are not significantly different (*p* > 0.05). (**a**) Triiodothyronine, juveniles; (**b**) triiodothyronine, adults; (**c**) thyroxine, juveniles; (**d**) thyroxine, adults.

Seasonal fluctuations in triiodothyronine and thyroxine concentrations are presented in Figure 4.

In the case of juvenile red king crabs, the October level of T3 was significantly higher when compared to the July level (Table 2). Adult red king crabs demonstrated similar concentrations of triiodothyronine in May, June, and October, while a significant increase in the T3 level was observed in August. In contrast, the concentrations of thyroxine were consistent throughout the study period (Table 2).

## 4. Discussion

The most abundant cohort in this study was adult males with 131–150 mm CL. This size class includes commercial-sized individuals, i.e., the most important population group in terms of its ecological role and significance for fishing. According to size-at-age data [36], the age of the males used for biochemical assays in this study varied from 3 to 16 years. The predominance of males has been reported by other authors at western sites of the Kola Peninsula [30], while females usually dominate the catch at eastern sites [33,35]. Adult females were less abundant in the larger size classes because their growth rate is lower than that of males, reflecting the fact that females invest more energy into reproduction processes than into somatic growth than males do [24,35].

Our study confirmed that both juvenile and adult individuals of *Paralithodes camtschaticus* have detectable concentrations of T4 and T3 in their hemolymph, but the present levels were higher than those reported by Zenzerov and Tipisova [40], probably due to the lower sample size used in the previous study and restricted study period. Iromo et al. [11] studied thyroxin concentrations in hemolymph of female mud crabs of *Scylla serrata* from Tarakan Island, East Borneo, Indonesia, and found that immature crabs exhibited the lowest concentration of T4 (8.50 ± 2.17 ng mL^–1^), maturing crabs have the intermediate level of T4 (11.23 ± 2.06 ng mL^–1^), and the highest concentration was found in ripe females (15.33 ± 7.20 ng mL^–1^). The same pattern was found for the ovaries of *Scylla serrata* (18.47 ± 2.06 vs. 27.07 ± 1.92 vs. 42.27 ± 3.17 ng mL^–1^), indicating that thyroxin plays an important role in vitellogenesis. Thyroid hormones have also been found in other invertebrate taxa. Huang et al. [12] reported that larvae of the Pacific oyster *Crassostrea* gigas contain triiodothyronine and thyroxine in concentrations of 0.1–0.35 µg g^–1^ and 0.5–4 µg g^–1^, respectively. They concluded that these oysters have the ability to synthesize thyroid hormones endogenously. Later radioimmunoassay analyses have shown the presence of T4 (concentration 3.49 ± 0.55 µg dL^–1^) in hemolymph of the giant African snail *Achatina fulica* [13]. The authors also found that the thyroxine level decreased significantly to 2.27 ± 0.16 µg dL^–1^ as a result of 4-week starvation. All these findings support the opinion that levels of thyroid hormones do significantly correlate to physiological conditions of animals, rising or falling in critical phases of their life cycles [1]. We found that adult red king crabs had lower concentrations of T4 than juvenile ones and an opposite pattern was observed for T3. Adult crabs are known to expend more energy due to larger size, reproduction, and greater migration activity [29]. As a result, the concentration of T3 in their hemolymph is higher than in juvenile crabs. Taking into account that this hormone is synthesized from T4, it is not surprising that the level of T4 was lower than in smaller crabs. A similar pattern (an increased level of T3 and a stable level of T4) has been reported for oysters of *Crassostrea gigas* [12].

Most likely, seasonal patterns observed for triiodothyronine concentrations in the hemolymph of the red crabs can also be attributed to fluctuations in their physiological conditions. Indeed, in juveniles, the highest concentration of T3 occurred in October. According to published data, juvenile red king crabs at age 3–5 years molt 2 or 3 times per year in March–May and October–December [29,43]. The molting process is a high-energy-dependent event and higher triiodothyronine levels are expected in the period when mass molting occurs. Although ecdysteroids are considered the major molting hormones in crustaceans [44], recent studies have indicated that molt induction in black tiger shrimps, *Penaeus monodon*, led to expression of genes associated with vitellogenin, including thyroid hormone receptor associated protein [45]. 

Adult red king crabs exhibited the highest concentration of T3 in August, i.e., the month when both large females and males start to migrate to the open sea. The first migration peak occurs in March–April when the majority of males leave coastal sites after mating and spawning. In October–December, the coastal area is occupied by overwintering individuals of *Paralithodes camtschaticus* [29]. Taking into account that triiodothyronine has important effects on mitochondrial energetics [46] and can modulate several metabolic events similar to the effects of vertebrate thyroids [4], the higher levels of T3 may be associated with more intense motor activity of adult red king crabs. Juvenile red king crabs seem to spend less energy due to their smaller size and weight and less active lifestyle [20,29].

Thyroid hormones have been shown to induce a plethora of physiologic actions in crustaceans. Thus, exogenous application of thyroxine led to a significant increase in growth and molt cycle events in postlarvae and early juveniles of the shrimp *Penaeus monodon*, with the maximum growth increments at a dose of 3 μg L^–1^ [47]. Roustaian and Gaik [48] also reported that exogenous application of T4 enhanced growth in larvae of the giant freshwater prawn *Macrobrachium rosenbergii* with the highest growth rates at doses of 1 and 2 μg L^–1^, but precocious molt promoting events were not observed. Iromo et al. [14] showed that maturation of female mud crabs *Scylla serrata* from vitellogenic stage 2 to 3 occurred 4 and 10 days earlier after injections of thyroxine at doses of 0.05 and 0.5 μg g^–1^, respectively, but the second dose resulted in 100% mortality of experimental animals. Further studies showed that injections of T4 promoted the development of embryos to hatching eggs depending on dosage, with the best results at 0.15 and 0.1 μg g^–1^ [15]. Adiputra et al. [49] have shown that injection of T4 at a dose of 0.1 μg g^–1^can induce a higher growth rate and better gonad development in male spiny lobsters of *Panulirus homarus*. Jayaprakas and Sambhu [50] found that thyroxine incorporated into a prepared diet promoted growth and feed utilization of white prawns of *Penaeus indicus*, being the most effective at 0.04 ppm. Recently, Pinandoyo et al. [51] have reported that T4 added to a diet for giant freshwater prawns stimulated their growth performance and decreased mortality rates, with the best results at a concentration of 10 mg kg^–1^ feed. Thus, thyroid hormones appear to promote growth and production in crustaceans.

Recently, Russian President Vladimir Putin has approved a strategy for the development of the Arctic zone of the Russian Federation for the period through 2035. Opportunities include further development of the aquaculture sector [27]. Coastal sites of the Kola Peninsula have favorable environmental conditions for invertebrate aquaculture [27,52], but profitable aquaculture cannot be organized without excellent knowledge regarding the ecology and physiology of target organisms [39,53,54]. The red king crab is a slow-growing species reaching the commercial size of 150 mm carapace width (or 137 mm carapace length) after 10 years of growth. Potential methods to decrease inter-molt intervals and improve growth performance of crustaceans include manipulation of their hormonal status by administration of steroid hormones [37,39,55] or double-stranded RNA (dsRNA) [56], dietary supplementation [50,51], and other methods [43]. Thyroid hormones may also be considered promoting agents for cultivation of *Paralithodes camtschaticus.* From this point of view, our results provide a baseline for further research in this field.

## 5. Conclusions

Although red king crabs lack a classical thyroid gland, they have the ability to utilize thyroid hormones which may be ingested exogenously, formed endogenously, or produced via catalysis (as has been suggested for echinoderms, mollusks, and mud crabs). Our data indicate that the hemolymph of adult crabs contains more triiodothyronine than juveniles, while immature crabs demonstrate higher thyroxine concentrations than mature individuals. Male and female red king crabs, both adults and juveniles, have similar concentrations of thyroid hormones. Seasonal changes in the triiodothyronine concentration reflect shifts in the physiological status of red king crabs associated with mass molting in juveniles and higher migration activity in adults. Further research should be focused on the exogenous administration of thyroid hormones to test their effects on growth rates, molting events, survival, and meat quality of red king crabs. Such studies would provide novel techniques for red king crab aquaculture in the Barents Sea.

## Figures and Tables

**Table 1:** Morphometric parameters of red king crabs used for biochemical analyses.

Sex	Maturity	N	Carapace Length, mm				Weight, g			
			X	SE	Min	Max	X	SE	Min	Max
M	Juv	7	66.8	4.3	54.6	83.1	239.7	47.7	131	432
M	Ad	29	130.7	4.3	85.6	162.7	2015.4	184.4	474	3805
M	Tot	36	118.3	5.6	54.6	162.7	1670.1	190.0	131	3805
F	Juv	16	71.5	3.8	49.0	91.7	293.3	40.5	97	532
F	Ad	11	120.1	5.1	99.8	158.0	1449.6	270.0	665	3820
F	Tot	27	91.3	5.6	49.0	158.0	764.4	156.2	97	3820
M + F	Juv	23	70.1	2.9	49.0	91.7	277.0	31.6	97	532
M + F	Ad	40	127.8	3.5	85.6	162.7	1859.8	156.4	474	3820

Note: M—male, F—female, Juv—juveniles, Ad—adults, tot—total, N—sample size, X—mean, SE—standard error, Min—minimum, Max—maximum.

**Table 2:** Results of ANOVA or Kruskal–Wallis tests (KWT) comparing the differences in thyroid hormone levels of red king crabs from the Barents Sea.

Hormone	Comparisons	Test	df	F (H)	*p*
T3	Juv-M vs. Juv-F	KWT	1	1.969	0.161
	Juv-M vs. Ad-M	KWT	1	**5.284**	**0.022**
	Juv-F vs. Ad-F	ANOVA	1	0.957	0.337
	Ad-M vs. Ad-F	ANOVA	1	3.393	0.073
	Tot-M vs. Tot-F	ANOVA	1	**6.970**	**0.011**
	Juv-Tot vs. Ad-tot	ANOVA	1	**10.950**	**0.002**
	July vs. Oct (Juv)	ANOVA	1	**4.540**	**0.045**
	May vs. June vs. Aug vs. Oct (Ad)	ANOVA	3	**9.480**	**<0.001**
T4	Juv-M vs. Juv-F	KWT	1	0.755	0.385
	Juv-M vs. Ad-M	KWT	1	1.122	0.290
	Juv-F vs. Ad-F	ANOVA	1	**7.099**	**0.013**
	Ad-M vs. Ad-F	ANOVA	1	0.345	0.560
	Tot-M vs. Tot-F	KWT	1	0.866	0.352
	Juv-Tot vs. Ad-tot	KWT	1	**7.750**	**0.005**
	July vs. Oct (Juv)	ANOVA	1	0.001	0.995
	May vs. June vs. Aug vs. Oct (Ad)	ANOVA	3	2.120	0.116

Note: T3—triiodothyronine, T4—thyroxine, M—male, F—female, Juv—juvenile, Ad—adult, tot—total, d—degree of freedom, F—F-ratio for ANOVA, H—chi-square for the Kruskal–Wallis test, *p*—probability level. Bold font indicates significant differences.

## Data Availability

The data are available on request from the corresponding author.
